# Application and improvement of YOLO11 for brain tumor detection in medical images

**DOI:** 10.3389/fonc.2025.1643208

**Published:** 2025-08-29

**Authors:** Weijuan Han, Xinjie Dong, Guixia Wang, Yuwen Ding, Aolin Yang

**Affiliations:** ^1^ School of Mechanical and Electronic Engineering, Zhongyuan Institute of Science and Technology, Zhengzhou, China; ^2^ Information and Communication Department, Henan Public Security Department, Zhengzhou, China; ^3^ Editorial Department, Henan Medical College, Zhengzhou, China; ^4^ School of Public Administration, Henan University of Economics and Law, Zhengzhou, China

**Keywords:** brain tumor, object detection, you only look once (YOLO), attention, intersection over union (IoU), mean average precision (MAP), giga floating point operations per second (GFLOPs)

## Abstract

Brain tumors pose a critical threat to human health, and early detection is essential for improving patient outcomes. This study presents two key enhancements to the YOLOv11 architecture aimed at improving brain tumor detection from MRI images. First, we integrated a set of novel attention modules (Shuffle3D and Dual-channel attention) into the network to enhance its feature extraction capability. Second, we modified the loss function by combining the Complete Intersection over Union (CIoU) with a Hook function (HKCIoU). Experiments conducted on a public Kaggle dataset demonstrated that our improved model reduced parameters and computations by 2.7% and 7.8%, respectively, while achieving mAP50 and mAP50–95 improvements of 1.0% and 1.4%, respectively, over the baseline. Comparative analysis with existing models validated the robustness and accuracy of our approach.

## 1 Introduction

Brain tumors present a serious risk to human health with potentially devastating consequences. Abnormal growth can interfere with brain function, causing severe neurological symptoms, cognitive impairment, and in many cases, mortality (1, 2). The classification of brain tumors serves as the foundation for clinical diagnosis, treatment planning, and prognostic assessment. The most authoritative international system is the World Health Organization (WHO) Classification of Tumors of the Central Nervous System, with the latest 5th edition (WHO CNS5) published in 2021 (3, 4). This classification integrates histopathology, molecular genetics, and clinical phenotypes to form an integrated diagnosis framework, replacing the previous morphology-based classification model. Based on tissue origin and biological characteristics, WHO CNS5 categorizes brain tumors into the following 6 categories: Neuroepithelial Tumors (Gliomas and Related Tumors), Meningeal Tumors, Cranial and Peripheral Nerve Tumors, Germ Cell Tumors, Sellar Region Tumors, and Metastatic Brain Tumors.

Tumor characteristics such as location, size, and grade are critical determinants of neurological impairments and functional deficits in patients with brain tumors. Location directly influences the specific deficits due to the brain’s functional specialization. For example, tumors in the motor cortex often cause contralateral limb weakness or paralysis, while lesions in the cerebellum may lead to ataxia and coordination difficulties. Size correlates with the severity of mass effect and peritumoral edema. Larger tumors (e.g., diameters >4 cm) exert greater mechanical pressure on surrounding tissues, causing midline shift, ventricular compression, and increased intracranial pressure, which manifest as headaches, nausea, altered consciousness, and even herniation. Grade reflects tumor aggressiveness and biological behavior. Low-grade tumors grow slowly and may remain asymptomatic for years, while high-grade tumors exhibit rapid infiltration, angiogenesis, and necrosis, leading to severe and progressive deficits. In summary, tumor location dictates the type of neurological deficits, size determines the extent of mass effect and increased intracranial pressure-related complications, and grade predicts the tempo and severity of clinical progression. Multidisciplinary management (5), including surgical planning, adjuvant therapies, and neurorehabilitation, must account for these interdependent factors to optimize outcomes.

Early and accurate detection of brain tumors is essential for treatment planning, as timely intervention can significantly improve patient prognosis and quality of life. Magnetic resonance imaging (MRI) (6) has become a primary diagnostic tool for brain tumors owing to its high soft-tissue contrast and detailed anatomical resolution. However, manual analysis of MRI scans for tumor detection is time-consuming and prone to error, relying heavily on medical expertise. Therefore, developing automated and reliable object-detection algorithms for brain tumors in MRI images has become a critical research priority.

Traditional machine learning algorithms to detect brain tumors in medical images, such as Haar cascades (7) and histograms of oriented gradients (HOG) (8) combined with support vector machines (SVM), have been applied to brain tumor detection. These methods depend on handcrafted features that require extensive domain knowledge and careful design. However, these methods often fail to generalize across datasets and imaging modalities, as performance is constrained by the complexity and variability of brain tumor appearance on MRI scans. The inability to extract high-level semantic information limits the accuracy and robustness of traditional machine-learning-based detection methods.

Deep learning has introduced transformative advances in object detection. Region-based convolutional neural networks (R-CNNs) (9), introduced by Girshick et al., marked a significant milestone by applying a data-driven approach to object detection. Faster R-CNN (10), an improved version of R-CNN, integrated a region proposal network (RPN), which reduces computational cost and increases detection speed while preserving accuracy. For brain tumor detection, Faster R-CNNs have demonstrated promise in accurately identifying tumor regions by leveraging deep convolutional features (11). However, its slow processing and complex two-stage architecture limit practical use in real-time medical diagnostics.

The single-shot multibox detector (SSD) (12) developed by Liu et al. has proven to be an efficient alternative to two-stage detectors. The model predicts the bounding boxes and class probabilities within a single network, enabling faster inference. By utilizing feature maps from different layers, an SSD can effectively detect objects of various scales, achieving a good balance between speed and accuracy. In brain tumor detection using MRI images, SSD has demonstrated the ability to detect tumors of different sizes; however, it still faces challenges in accurately detecting small and irregularly shaped tumors because of the limited receptive field of shallow layers and loss of spatial information in deeper layers.

The You Only Look Once (YOLO) series (13), introduced by Redmon et al., has attracted wide attention for its significant advantages in object detection. Firstly, the single-stage architecture of YOLO endows it with high computational efficiency, and is capable of real-time or near-real-time detection. This is highly valuable in clinical settings, where rapid results help doctors make timely diagnostic decisions. For example, in the context of brain tumor detection from MRI images (14), doctors can promptly access results, and quickly specify examinations or treatment. Secondly, YOLO captures global contextual information from the entire input image. In contrast to other methods that focus on local regions separately, the holistic approach of YOLO helps better understand the relationships between different parts of an image. Simultaneously, YOLO can accurately identify the location and category of tumors, even when they have complex shapes. This holistic understanding is particularly valuable for addressing the complexity of brain tumors in MRI scans. Moreover, the YOLO series has demonstrated strong generalization across different datasets and scenarios such as COCO (15), PASCAL VOC2012, NEU-DET, RSOD (16), LOCO dataset (17), Figshare dataset (18), and so on. With continuous improvements in its architecture and training strategies over successive versions (19–22), it can adapt well to the variations in image quality, tumor appearance, and imaging parameters commonly encountered in real-world medical imaging applications. This adaptability renders YOLO a reliable tool for detecting brain tumors in varied MRI datasets.

The original YOLO can achieve real-time performance on standard graphics processing units (GPUs), rendering it suitable for applications requiring rapid detection. Subsequent versions of YOLO, such as YOLOv5, YOLOv8, and beyond, have continuously improved the architecture and introduced advanced techniques, further enhancing detection performance.

This study focuses on YOLOv11 (23), an iteration of the YOLO series released in 2024. Building on the achievements of its predecessors, YOLOv11 integrates advanced architectures and optimization strategies to overcome limitations in handling the complex and diverse characteristics of brain tumors in MRI images. Given the increasing demand for efficient and accurate brain tumor detection in clinical practice, YOLOv11 holds considerable potential for achieving superior performance in terms of detection speed, accuracy, and the ability to identify tumors of various shapes and sizes. This study aimed to explore the capabilities of YOLOv11 in brain tumor detection from MRI images and conduct comprehensive experiments to evaluate its effectiveness using a publicly available Kaggle dataset.

The structure of this paper is organized as follows: Section 2 describes the related work. Section 3 provides a detailed description of our methodology and improvement measures. Section 4 presents the experimental results, a comprehensive performance analysis and comparison with other models. Section 5 provides an overall discussion. Finally, Section 6 concludes the paper.

## 2 Related work

In recent years, numerous studies have been conducted on the detection of brain tumors in MRI images using deep learning algorithms, particularly the YOLO series algorithms, which have demonstrated excellent performance.

Kharb et al. (24) proposed a hybrid model for brain tumor classification that combined faster R-CNN and EfficientNet. The hybrid model achieved a notable accuracy of 98.96% during the training phase and 99.2% during the testing phase on the Figshare (25) Datasets.

Hikmah et al. (26) introduced a novel approach for precise brain tumor detection, combining various approaches such as morphological operations for tumor segmentation, image enhancement, and a deep learning architecture based on MobileNetV2-SSD with feature pyramid network (FPN), where the FPN level originally set to 3 had been modified to level 2, which enhanced the detection of smaller objects. The proposed model obtained a recall value of around 98% and a precision value of around 89%.

Alsufyani (27) explored the use of several deep-learning models, including YOLOv8, YOLOv9, Faster R-CNN, and ResNet18, for the detection of brain tumors from MRI images. The results on the Kaggle’s Medical Image Dataset for Brain Tumor Detection, consisting of 3903 brain MRI images, demonstrate that YOLOv9 outperforms the other models in terms of mAP (0.826) and accuracy (0.784), highlighting its potential as the most effective deep-learning approach for brain tumor detection.

Chen et al. (28) proposed the YOLO-NeuroBoost model, combining the improved YOLOv8 algorithm with innovative techniques, such as the dynamic convolution kernel warehouse, attention mechanism CBAM, and inner-GIoU loss function. It achieved mean average precision (mAP) scores of 99.48% and 97.71% on the BR35H (29) and RoboFlow (30) datasets. High mAP scores indicate the high accuracy and efficiency of the model in detecting brain tumors in MRI images. However, the model has more parameters and GFLOPs than YOLOv11, resulting in a larger model size.

Kang et al. (31) proposed PK-YOLO, which included the following three components: a pretrained, pure lightweight CNN-based backbone via sparse masked modeling, a YOLO architecture with a pretrained backbone, and a regression loss function for improving small object detection. PK-YOLO achieved a mAP of 58.2% on the BR35H dataset.

Monisha and Rahman et al. (32) proposed a federated learning architecture to enhance brain tumor detection by incorporating the YOLOv11 algorithm. The federated learning approach safeguards patient data while enabling collaborative deep-learning model training across multiple institutions. On a synthetic brain tumor dataset with about 10,000 MRI images, the model achieved a mean average precision (mAP) of 90.8% and an mAP50–95 of 65.3%.

Dulal et al. (33) proposed an enhanced version of YOLOv8. Their work significantly advances automated brain tumor detection by introducing an improved YOLOv8 model. Through strategic modifications, including the integration of a Vision Transformer block, Ghost Convolution, and RT-DETR, their model achieved 91% mAP0.5 on a public Kaggle dataset.

Wahidin et al. (34) used several of the latest versions of the YOLO model, namely YOLOv11m, YOLOv10m, YOLOv9m, and YOLOv8m, to detect brain tumors such as gliomas, meningiomas, and pituitary tumors in MRI images. Hyperparameter tuning was conducted using the Bayesian optimization and HyperBand (BOHB) search algorithm with ray tuning through 16 trials. YOLOv11m achieved the highest accuracy, with an mAP50 of 0.934 and an inference speed of 70.550 FPS. In contrast, YOLOv8m delivered the fastest inference speed of 80.471 FPS.

Bai et al. (35) proposed the SCC-YOLO architecture, integrating the SCConv module into YOLOv9. The SCConv module improves convolutional efficiency by reducing spatial and channel redundancy and enhancing image feature learning. This study examined the effects of different attention mechanisms with YOLOv9 on brain tumor detection using Br35H and custom datasets. The results indicate that SCC-YOLO improves mAP50 by 0.3 to 95.7% on the BR35H dataset and by 0.5 to 86% compared with YOLOv9. SCC-YOLO demonstrated strong performance in brain tumor detection.

This study involved two primary improvements. First, the YOLOv11 network architecture was enhanced by integrating several newly designed attention modules to strengthen the feature extraction capabilities of the network. Second, the loss function was modified to increase the loss value of low-quality prediction boxes, and promote rapid convergence of the model.

## 3 Materials and methods

The YOLO series of algorithms has demonstrated strong performance in detecting brain tumors in MRI images, particularly in terms of accuracy and efficiency. However, the algorithms may have different performances in different datasets and application scenarios, and further research and improvements are needed to improve the accuracy and efficiency of brain tumor detection and to serve clinical diagnosis better.

### 3.1 YOLOv11

**Figure 1:**
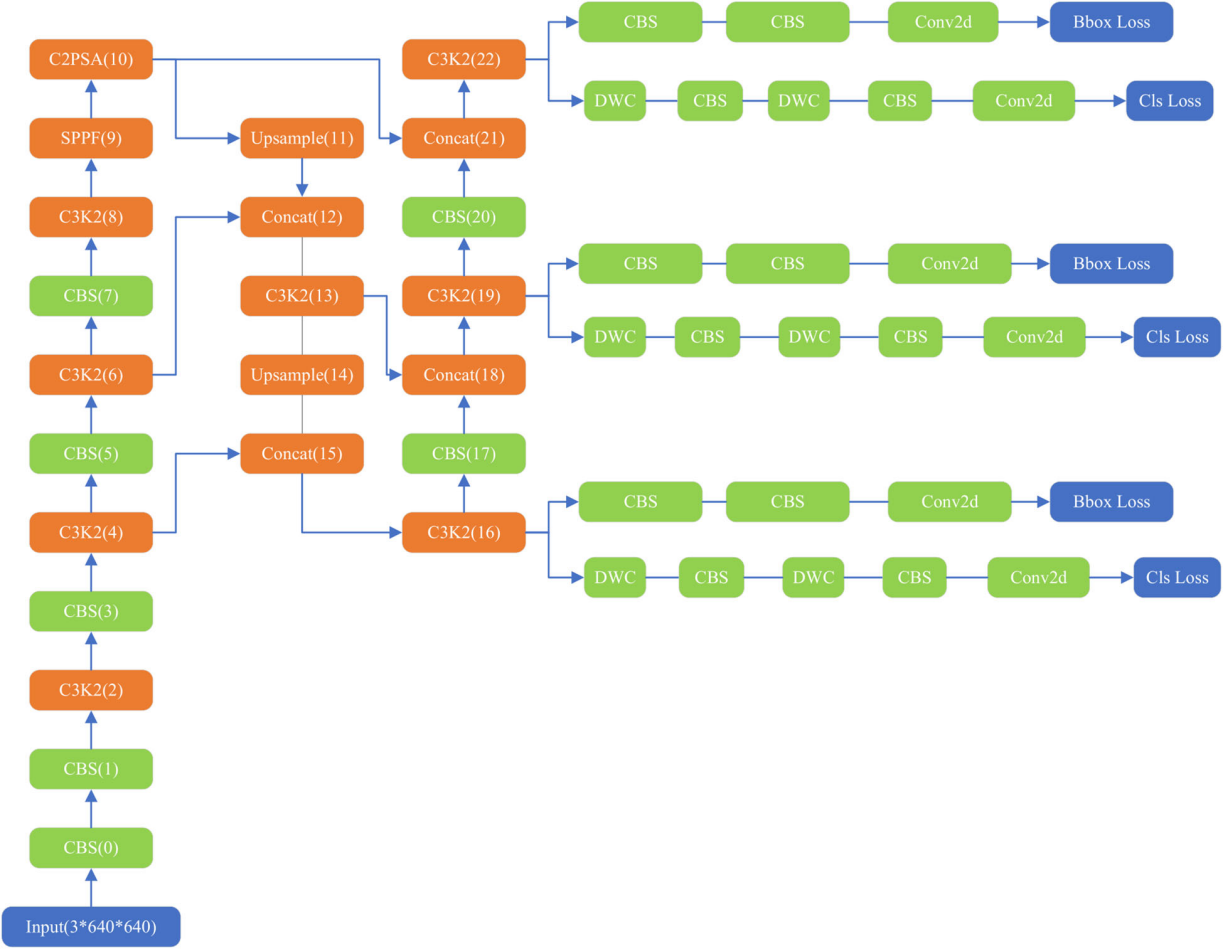
Structure of original YOLOv11.

The YOLOv11 structure (**Figure 1**) comprises three main components: the backbone, neck, and head (36, 37). The backbone contains 0–10 convolution modules, the neck layer comprises 11–22 parts, and the rest are three parallel detection heads that detect feature maps of 20 × 20, 40 × 40, and 80 × 80, and generate 8,400 possible detection results.

As the core of feature extraction, the backbone of YOLOv11 replaces YOLOv8’s C2f module with an improved C3K2 module and standard convolution (CBS). C3K2 module uses multi-scale convolution kernel C3K, where K is an adjustable convolution kernel size, such as 3 × 3, 5 × 5, etc. This design can expand the receptive field, allowing the model to capture a wider range of contextual information, especially suitable for large object detection or scenes with complex backgrounds. The CBS module mainly consists of three parts: Conv (convolution layer), BN (Batch Normalization) and SiLU (activation function). It also adds a C2PSA (Cross-Level Pyramid Slice Attention) module after SPPF, enhancing global feature modeling capabilities through a multi-head attention mechanism. This design enables the network to more effectively capture long-range dependencies, which is particularly important for occluded objects and complex scenes. The Feature Pyramid Network (FPN) structure is retained at the neck layer. The neck layer also uses C3K2 and CBS convolutions for extraction, with feature fusion performed using the Concat operation. The head layer, like previous versions, also includes three detection heads. Each head employs depthwise separable convolution (DWC) and standard convolution (CBS).

YOLOv11’s loss function continues the YOLO series’ pursuit of a balance between detection accuracy and speed. Targeted at the decoupled head structure, the loss function is divided into three parts: bounding box regression loss, confidence loss and classification loss, Bounding box regression loss enables the model to accurately locate the target, confidence loss can optimize the accuracy of the prediction box and improve the model’s ability to judge whether the target exists in the prediction box, and classification loss determines the category of the image in the prediction box. Bounding box regression includes the CIoU (Complete Intersection over Union) (38) loss and the DFL (Distribution Focal Loss) (39), which take into account the overlap, position, and shape of the bounding boxes. The total loss is a weighted sum of these three losses. The loss function calculation formula is shown in Equations 1, 2. In the equations, *L_box_* represents bounding box regression loss, *L_obj_* represents confidence loss, *L_cls_* represents classification loss, *L_CIoU_* represents CIoU loss, *L_DFL_* represents DFL loss and *α*, *β*, and *γ* represent weight parameters.


(1) $$L_{total}=\;\alpha L_{box}+\;\beta L_{obj}+\gamma L_{cls}$$

(2) $$L_{box}=L_{CIoU}+L_{DFL}\;$$

### 3.2 Main methods

Due to hardware limitations in clinical application environments and the demand for faster speeds, we are committed to reducing the number of model parameters and computational complexity, and improving detection accuracy. we integrated a set of novel attention modules into the network. This study replaces the original self-attention module C2PSA with a newly designed spatial attention module. At the same time, this study uses an improved loss function instead of the original loss function CIoU.

### 3.3 Attention

This study employed three attention mechanisms: Spatial attention, Shuffle3D attention, and Dual-channel attention. The latter two are newly designed attention mechanisms.

#### 3.3.1 Shuffle3D attention

**Figure 2:**
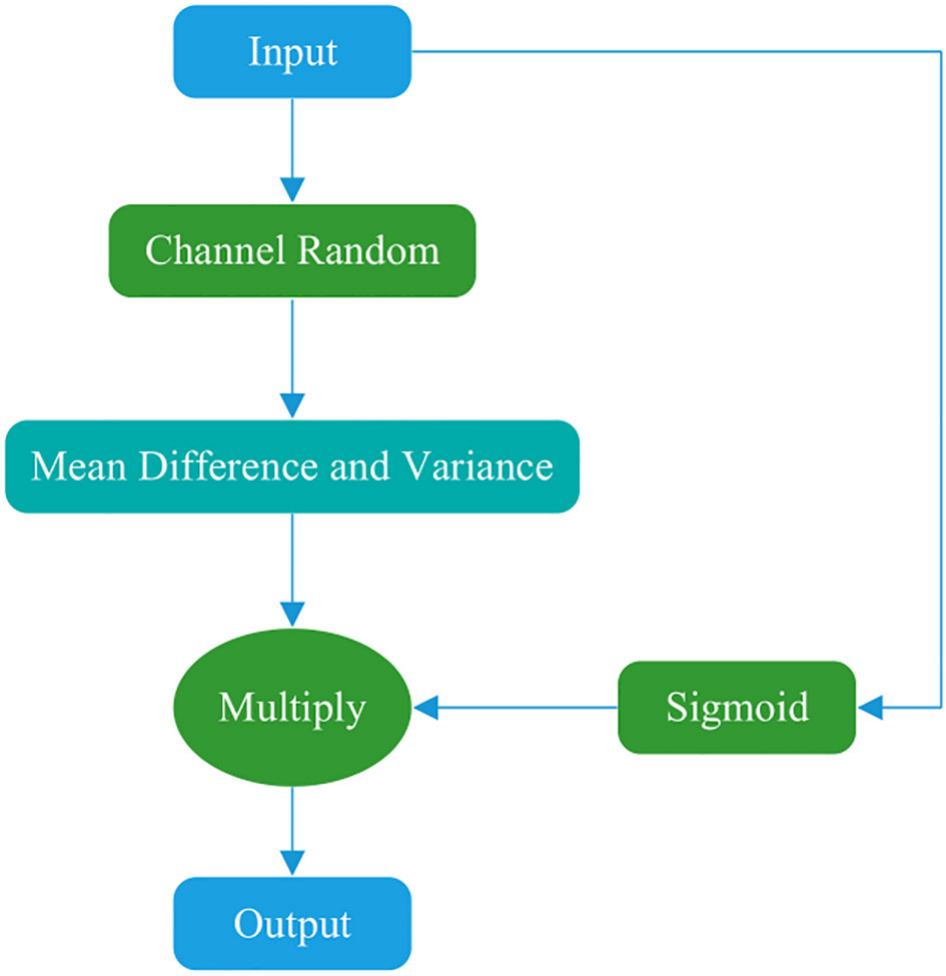
Shuffle3D attention structure.


This study draws on the concepts of the Shuffle (40) and SimAM (41) attention mechanisms to propose a novel attention mechanism, designated as Shuffle3D (**Figure 2**). On the one hand, channel rearrangement is applied to disrupt the original channel order, introducing random diversity and enabling joint modeling of different features. This module increases information exchange and balance between channels. On the other hand, a spatial inhibition mechanism is used. In neuroscience, information-rich neurons often exhibit different discharge patterns from the surrounding neurons. Moreover, activated neurons commonly inhibit neighboring neurons. Thus, neurons exhibiting spatial inhibition should receive greater emphasis. The calculation formulae of inhibition effects are presented in Equations 3–5, where *x* represents the input feature map, *x_ij_* represents a point in the feature map, *e* represents the mean, *H* represents the height of the feature map, *W* represents the width of the feature map, *u* represents the degree of deviation from the mean at a certain point on the feature map, and *α* and *β* are the regulators, which are set to the -4th power of 10 and 0.5, respectively. Neurons that deviate more from the mean yield higher activation function values.

(3) $$e\;=\;\frac{1}{H*W-1}\sum_{j=1}^{H}\sum_{i=1}^{W}x_{ij}$$

(4) $$u=\frac{{\left(x-e\right)}^{2}}{4\left(\sum_{j=1}^{H}\sum_{i=1}^{W}x_{ij}/\left(H*W-1\right)+\alpha \right)}+\beta$$

(5) x=sigmoid(u)*x

#### 3.3.2 Spatial attention

**Figure 3:**
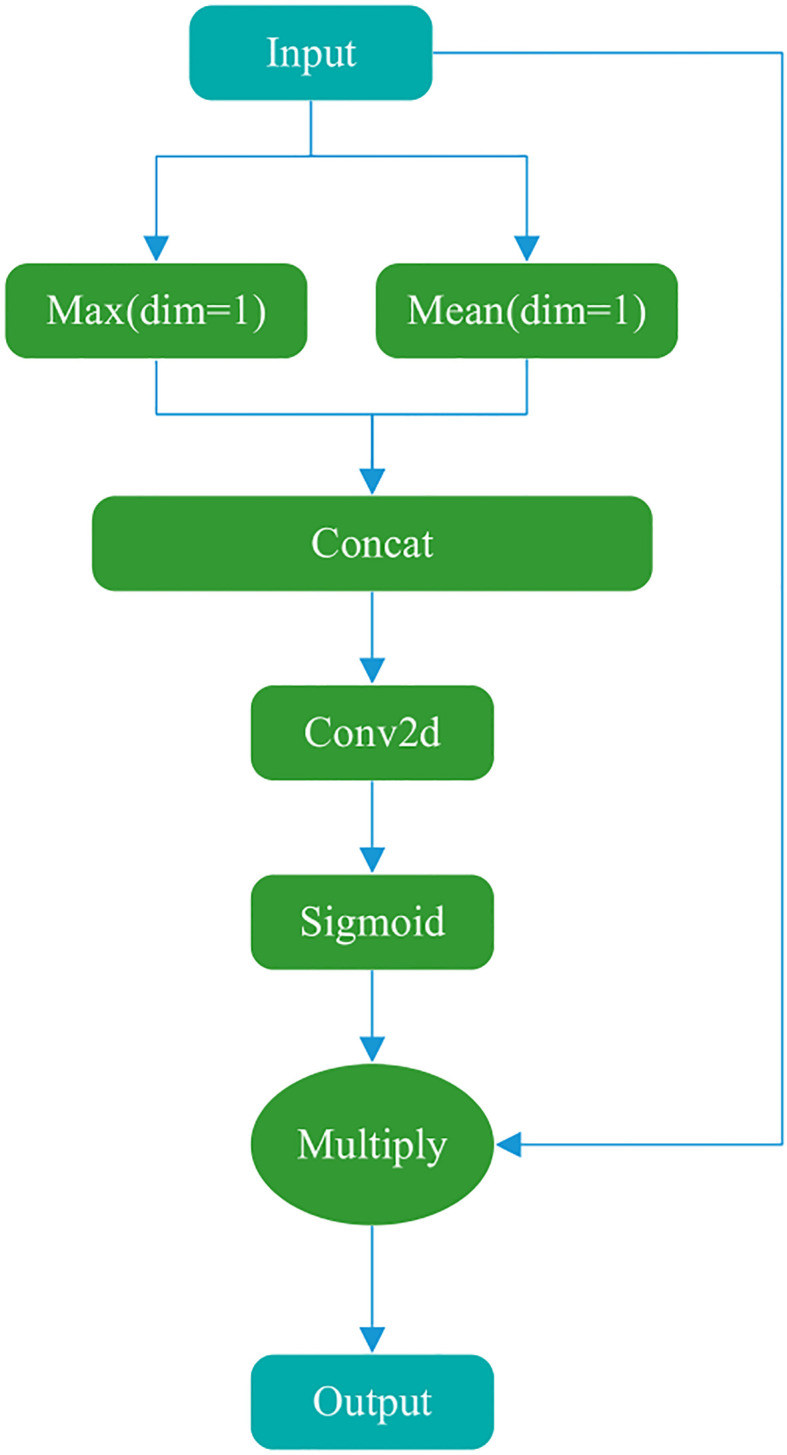
Spatial attention structure.

The main goal of the Spatial attention module (**Figure 3**) is to explicitly model the dependencies between spatial locations and generate a spatial attention map. First, the input features are max-pooled and average-pooled in the channel dimension to generate two spatial descriptors. These two spatial descriptors are then concatenated in the channel dimension and passed through a convolutional layer to generate a spatial attention map. Finally, the values of the spatial attention map are normalized to the range (0, 1) using a sigmoid function and multiplied by the input tensor to generate the output.

The Conv2d module in the figure uses a kernel of (7,7), a stride of 1, padding of 3, 2 input channels, and 1 output channel (number of filters). These parameters ensure that the spatial dimensions (w, h) of the input and output feature maps are consistent and combine the results of average pooling and max pooling.

#### 3.3.3 Dual-channel attention

**Figure 4:**
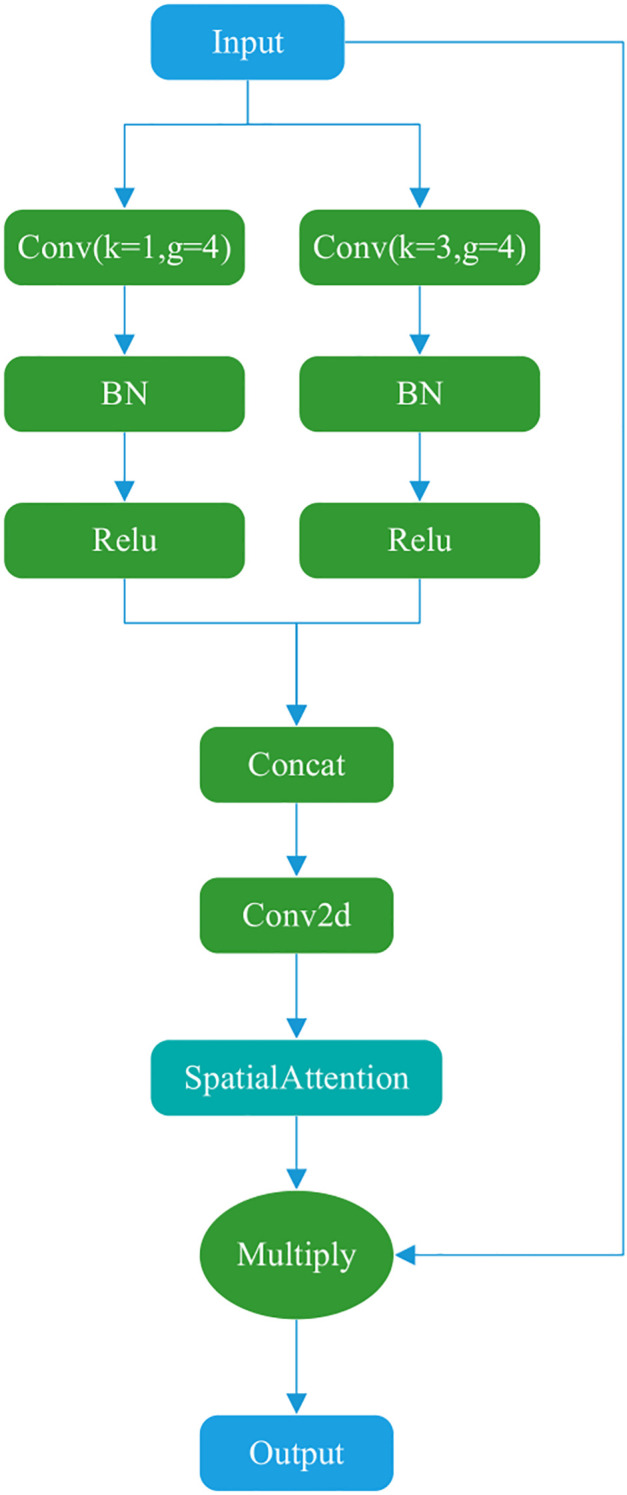
Dual-channel attention structure.

**Figure 4** illustrates the Dual-channel attention, which comprises two main components. The Dual-channel attention borrows the idea of parallel convolution of different sizes of kernels from Inception (42). The first part uses two parallel convolution operations with different convolution kernel sizes to capture additional feature information. The second part involves concatenation, convolution, and spatial attention computation. The final result is multiplied by the input to produce the output.

#### 3.3.4 New structure of the YOLOv11 networks

**Figure 5:**
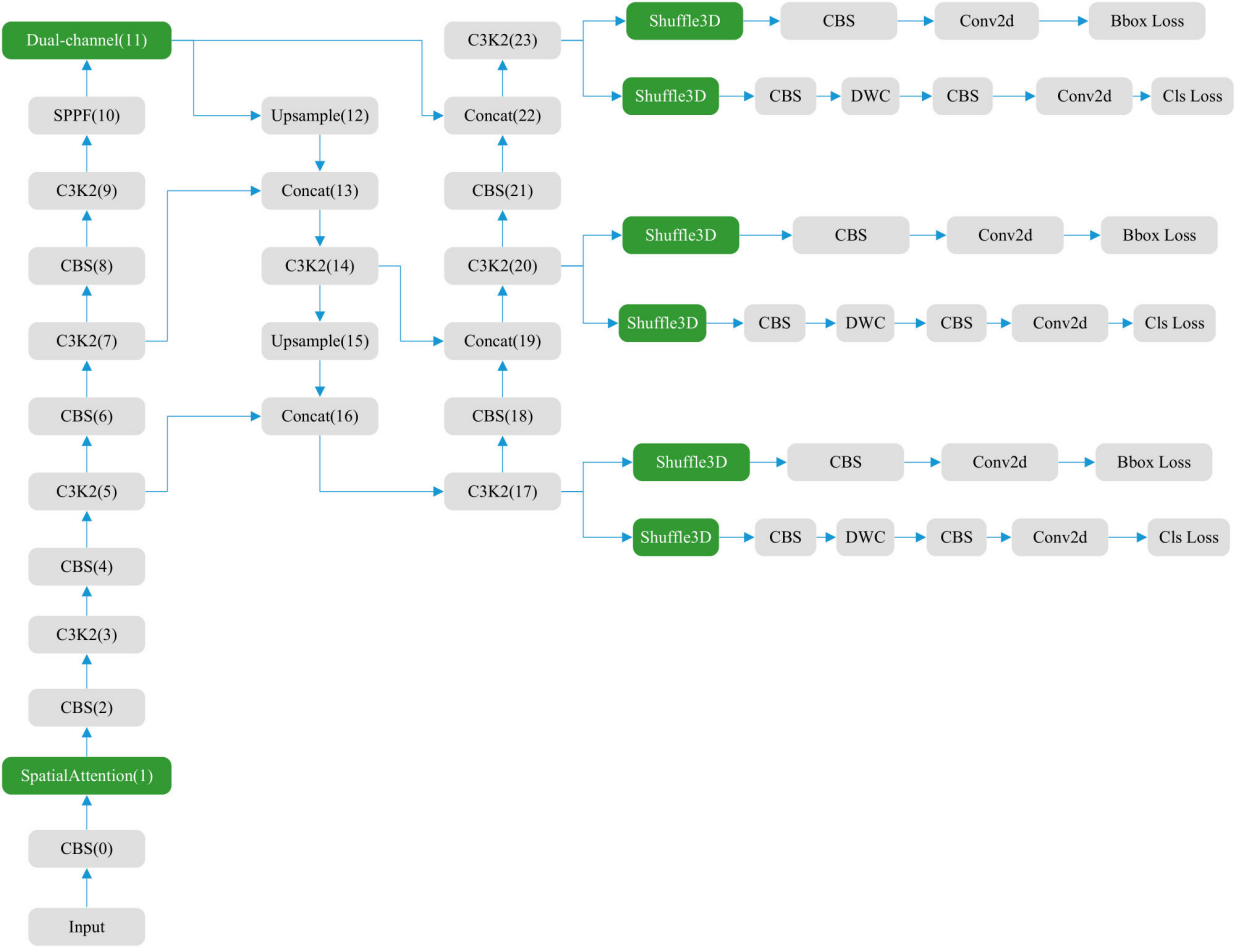
New structure of YOLOv11 with attention.

To enhance feature extraction in convolutional neural networks, we integrated the newly designed Shuffle3D with Spatial and Dual-channel attention. The positions of the attention modules are shown in **Figure 5**. The blue areas represent attention modules that are newly added or that replace the original ones. Dual-channel replaces the original self-attention module C2PSA, greatly reducing the computational load. Shuffle3D replaces the first CBS and DWC convolution modules on each detection head, enhancing the ability of the model to extract features from key regions.

### 3.4 HKCIoU

In the original YOLOv11, complete intersection over union (CIoU) serves as the boundary regression loss function, as shown in Equations 6–8. The *CIoU* loss refers to the loss during training and validation. The *IoU* stands for Intersection over Union. The *ρ* represents the distance between the center points of the predicted box and the true box, and *c* represents the diagonal distance of the minimum closure area that can contain both the predicted and true boxes. *b^p^* and *b^t^* represent the center points of the predicted box and the true box respectively. *w^t^* represents the width of the true box, and *w^p^* represents the width of the predicted box. *h^t^* represents the height of the true box, and *h^p^* represents the height of the predicted box. CIoU adds the penalty term of *α* and *β*, which are parameters used to measure the consistency of the aspect ratio.


(6) $$CIoU\;=\;IoU-\frac{\rho^{2}\left(b^{p},b^{t}\right)}{c^{2}}-\alpha *\beta$$

(7) $$\beta =\frac{4}{\pi^{2}}{\left(arctan\frac{w^{t}}{h^{t}}-arctan\frac{w^{p}}{h^{p}}\right)}^{2}$$

(8) $$\alpha =\frac{\beta }{1-IoU+\beta }$$

**Figure 6:**
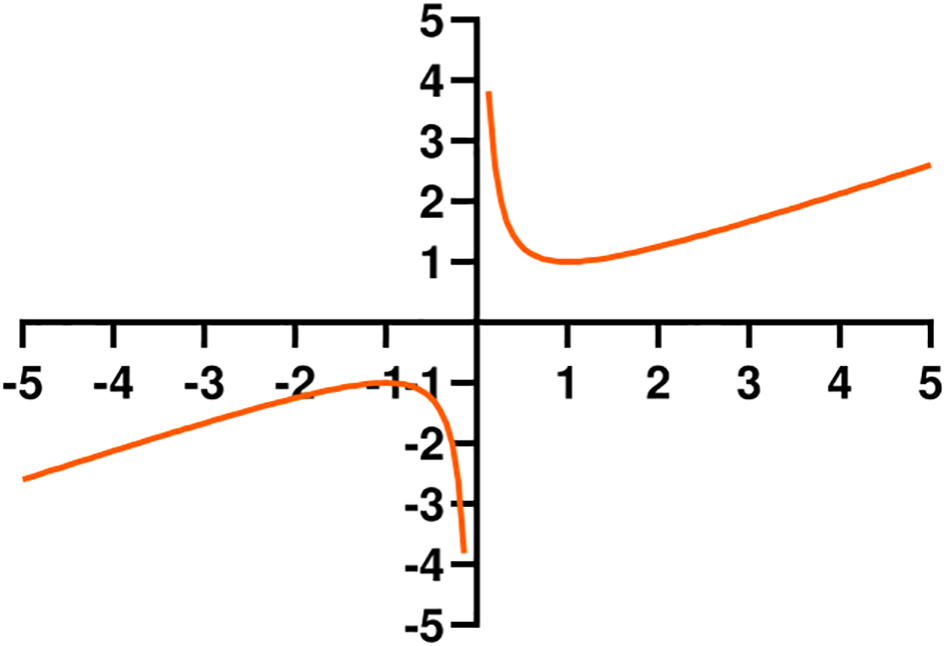
Hook function.


The hook function opens upward in the first quadrant (**Figure 6**). It is used to adjust the CIoU value, forming the HKCIoU. For a smaller CIoU, the loss is relatively amplified, and for a larger CIoU, the loss is relatively reduced, thereby accelerating the network convergence and enabling the network parameters to reach the optimal value faster. The calculation is given in Equations 9, 10. x represents the loss of CIoU, *a* and *b* are hyperparameters. *a* and *b* are both set to 0.5 where the value of equation has reached the minimum when *x* equals 1.

(9) $$f\left(x\right)=\;ax+\frac{b}{x}\;\left(ab>0\right)$$

(10) $$HKCIoU=\left(a*CIoU+\frac{b}{CIoU}\right)*CIoU\;$$

## 4 Results

The experimental hardware setup includes a 13th Gen Intel(R) Core(TM) i5-13600KF, 3500 MHz, 14 cores, 32 GB of RAM, and an RTX 4060Ti GPU with 16 GB of VRAM. The software environment included Windows 11, Python 3.8, Torch 1.13.1, CUDA 11.7, and PyCharm 2021.3. Each model was trained for 100 epochs, with a batch size of 32. The model employed SGD as the optimizer, with an initial learning rate of 0.01, a momentum of 0.937, and a weight decay of 0.0005.

YOLOv11 extensively utilizes various data augmentation techniques in training, including but not limited to HSV adjustment (hue, saturation, brightness transformation), random flipping/rotation, scaling, geometric affine transformation, random erasure, and Mosaic enhancement, significantly improving the model’s adaptability to scale changes, occluded scenes, and small targets. YOLOv11 closes Mosaic at the end of training and switches to standard image training in the last 10 epochs to avoid overfitting caused by differences in distribution between synthesized images and real data.

A Brain Tumor Detection Dataset (43) from Kaggle was used as experimental data. The dataset contains 5,249 MRI images divided into training and validation sets. The training set consists of 4,737 images, including 1,153 Glioma, 1,449 Meningioma, 711 No Tumor, and 1,424 Pituitary images. The validation set consists of 512 images, including 136 Glioma, 140 Meningioma, 100 No Tumor, and 136 Pituitary images. Each image was annotated with YOLO-format bounding boxes and labeled with one of four brain tumor classes. The evaluation indicators of the model include parameter count, computational complexity, mAP50, mAP50-95, and FPS (Frames Per Second).

### 4.1 Attention ablation experiment

In the experiment, we used three attention mechanisms, and the ablation results of the three attention mechanisms are shown in **Table 1**. From the table, it can be seen that the use of attention mechanism resulted in varying degrees of increase in mAP indicators. Compared to the model numbered 8, the models numbered 2, 3, and 5 achieved higher performance, but their parameter and computational complexity increased significantly. Although the parameter quantity and computational complexity of models numbered 4, 6, and 7 are lower than model 8, their mAP indicators are not as good as model 8. Their results are very close, and there is some fluctuation in the results of different experiments in the same model. Taking all factors into consideration, we have chosen to use the model 8 with three types of attention, namely Spatial, Dual-channel, Shuffle attention.

**Table 1:** Attention ablation experiment based on YOLOv11n.

Number	Attention	Parameters (million)	GFLOPs	mAP50 (%)	mAP50-95 (%)
1	YOLOv11n	2.59	6.4	95.8	78.1
2	+Spatial	2.59	6.5	96.5	79.7
3	+Dual-channel	2.64	6.5	96.3	78.7
4	+Shuffle3D	2.47	5.8	96.4	78.4
5	+Spatial +Dual-channel	2.64	6.5	96.7	79.5
6	+Spatial +Shuffle3D	2.47	5.8	96.3	78.6
7	+Dual-channel +Shuffle3D	2.52	5.8	96.5	78.5
8	+ALL	2.52	5.9	96.5	78.6

### 4.2 Ablation experiment

**Figure 7:**
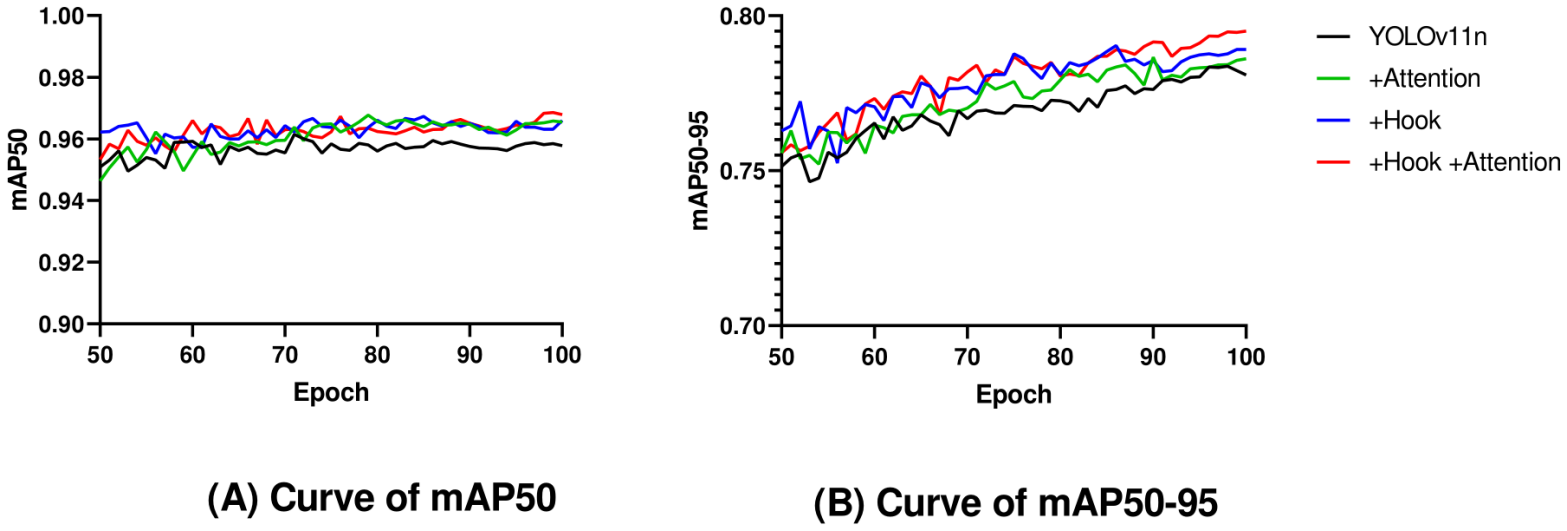
Curves of mAP50 and mAP50–95 with epoch in ablation experiments.

**Figure 8:**
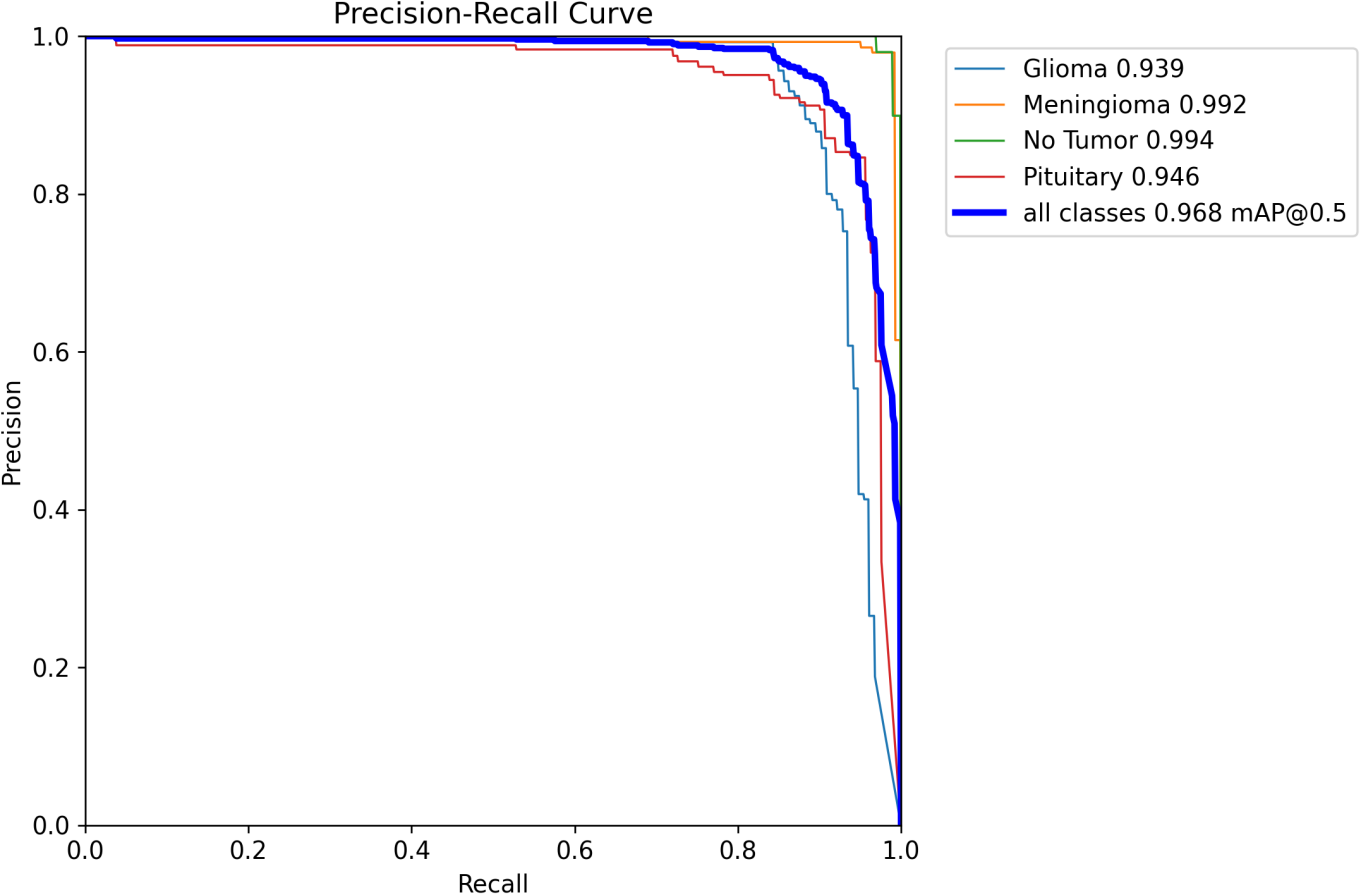
PR curve of YOLOv11n-HA on test set.

Ablation experiments (**Table 2**, **Figure 7**) demonstrated that when only the hook function was used, both mAP50 and mAP50–95 were improved by 0.8%. When only the attention mechanism was used, mAP50 and mAP50–95 were improved by 0.7% and 0.5%, respectively. The model using both Hook and Attention, named YOLOv11n-HA, improved mAP50 and mAP50–95 by 1% and 1.4%, respectively, with a 2.7% reduction in parameters and a 7.8% reduction in calculations. Simultaneously, in terms of FPS, YOLOv11n-HA achieved a 1.5% rise compared to the baseline model. The PR curve of YOLOv11n-HA on the test set is shown in **Figure 8**, which includes the mAP50 values of each subclass.

**Table 2:** Improved ablation experiment based on YOLOv11n.

Model	Parameters (million)	GFLOPs	mAP50 (%)	mAP50-95 (%)	FPS (f/s)
YOLOv11n	2.59	6.4	95.8	78.1	66.91
+Hook	2.59	6.4	96.6	78.9	65.96
+Attention	2.52	5.9	96.5	78.6	67.85
+Hook +Attention (YOLOv11n-HA)	2.52	5.9	96.8	79.5	67.90

To demonstrate the robustness of the model, we conducted three experiments on the final model, YOLOv11n-HA, which includes two improvements. The results are shown in **Table 3**. From the table, it can be seen that there is some fluctuation in the results of the model. This study speculates that this phenomenon is not only related to the jitter of the neural network but also to the random channel rearrangement of Shuffle3D attention, which increases the randomness of the model. Based on the mAP50 metric, we selected the experiment with the median value as the result. That is the one with an mAP50 value of 96.8%.

**Table 3:** Results of three experiment based on YOLOv11n-HA.

Number	mAP50 (%)	mAP50-95 (%)
1	96.7	79.1
2	96.8	79.5
3	96.9	79

### 4.3 Comparison

**Table 4** presents results comparing YOLOv11n-HA with other models, including non-YOLO and YOLO series deep learning models. The models and data involved were retrained and validated using the same dataset for this study.

**Table 4:** Comparison results with other state-of-the-art models used in the detection of brain tumors.

Model	Parameters (million)	GFLOPs	mAP50 (%)	mAP50-95 (%)
Faster-RCNN (ResNet50)	28.30	470.48	91.2	59
SSD (VGG)	24.01	61.06	93.7	70.7
YOLOv5n	2.18	5.9	96.3	78.1
YOLOv8n	2.69	6.9	96.2	79
YOLOv9s	6.32	22.7	96.4	79.7
YOLOv10n	2.71	8.4	95.4	78.4
RT-DETR (L)	32.8	108.0	92.9	71.6
YOLOv11n-HA	2.52	5.9	96.8	79.5

#### 4.3.1 Comparison with non-YOLO series

Faster-RCNN and SSD not only have lower mAP50 and mAP50–95 indicators than YOLOv11n-HA but also have several times more parameters and computational complexity. Compared with RT-DETR(L), YOLOv11n-HA uses only 7.7% of the parameters and 3.3% of the computational complexity, while achieving increases of 3.9% in mAP50 and 7.9% in mAP50-95.

#### 4.3.2 Comparison with YOLO series

Comparing the metrics of YOLOv11n-HA with that of YOLOv5n, we observe that the GFLOPs of YOLOv11n-HA remain the same, the number of parameters increases by 15.6% from 2.18M to 2.52M, and the mAP50 and mAP50–95 indicators increase by 0.5% and 1.4%, respectively. Compared with that of YOLOv8n, the number of parameters in YOLOv11n-HA decreased by 6.3%, computational GFLOPs decreased by 14.5%, and the mAP50 and mAP50–95 indicators increased by 0.6% and 0.5%, respectively. Compared to that of YOLOv9s, the number of parameters of YOLOv11n-HA decreased by 60.1%, the number of calculations decreased by 74%, mAP50 increased by 0.4%, and mAP50–95 decreased by 0.2%. Under the condition of a significant decrease in the number of parameters and the cost of calculations, YOLOv11n-HA is still better than YOLOv9s in terms of mAP50. Compared with that in YOLOv10n, the number of parameters in YOLOv11n-HA decreased by 7.0%, computational GFLOPs decreased by 29.8%, and the mAP50 and mAP50–95 indicators increased by 1.4% and 1.1%, respectively.

## 5 Discussion

This study introduces two key improvements to the original YOLOv11 model. First, it improves the YOLOv11 network structure by adding the Spatial attention, two newly designed Shuffle3D attention schemes, and Dual-channel attention. Second, it improves the loss function by introducing a hook function to adjust the CIoU loss, amplify penalties for low-quality predictions, and accelerate network convergence. The ablation experiment proved that, compared with native YOLOv11n, YOLOv11n-HA increased mAP50 and mAP50–95 by 1% and 1.4%, respectively, while the model parameters and computational GFLOPs decreased by 1.4% and 2.7%, respectively. Compared to other state-of-the-art models, YOLOv11n-HA achieved a superior recognition rate.

**Figure 9:**
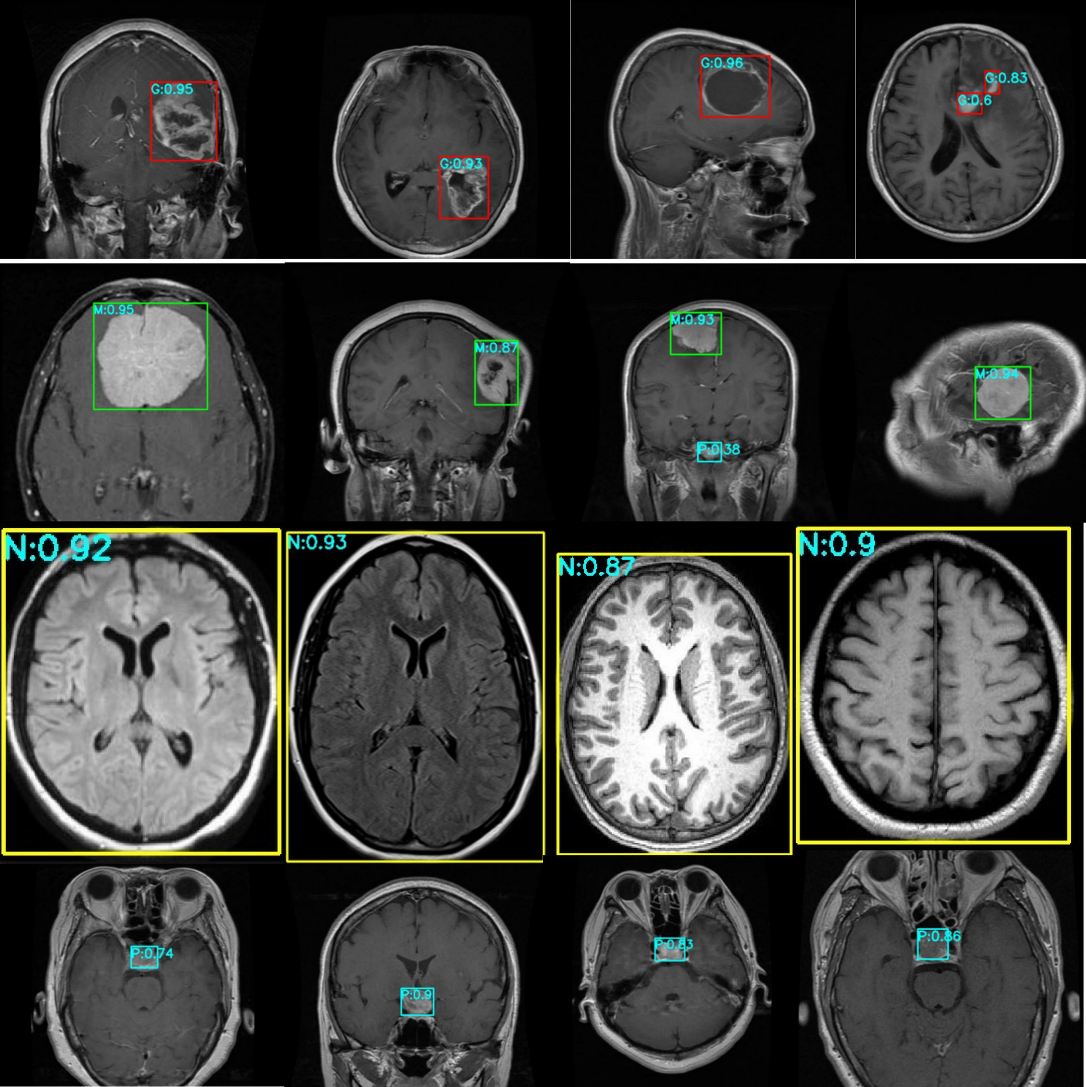
Effect diagram of brain tumor detection on the dataset.

**Figure 9** presents the test results for the Kaggle brain tumor dataset. The red box and G represent Glioma, the green box and M represents Meningioma, the yellow box and N represent No tumor, the cyan box and P represents Pituitary. The numbers behind represent the probability value of belonging to this class.

This study makes a significant contribution to the literature because it introduces a lightweight, computationally efficient model that achieves superior detection performance compared to state-of-the-art methods, thereby offering a practical solution for clinical applications with hardware constraints.

Further, this study addresses a critical challenge in medical imaging, accurate and rapid detection of brain tumors, by combining deep learning innovations with clinical relevance, offering insights that bridge technical development and healthcare impact. The proposed model achieves a strong balance between detection performance and computational efficiency, making it especially suitable for clinical deployment where hardware limitations exist. By providing accurate, real-time tumor localization in MRI images, this work contributes toward scalable and practical AI-assisted diagnostic solutions for healthcare settings.

## 6 Conclusion

This study used YOLOv11n to detect brain tumors in a public MRI dataset from Kaggle and introduced two key improvements. The first enhanced the network structure by integrating attention mechanisms, namely Shuffle3D attention and Dual-channel attention, which are newly designed in this study. The second introduces a new loss function, HKCIoU, which amplifies the loss for poorly predicted boxes via the hook function to accelerate network convergence. Ablation experiments demonstrate that mAP50 increased to 96.8% and mAP50–95 to 79.5%, with a 2.7% decrease in the number of parameters and a 7.8% decrease in GFLOPs.

## Data Availability

The original contributions presented in the study are included in the article/supplementary material. Further inquiries can be directed to the corresponding author.
